# Reliability of Imageless Computer-Assisted Navigation for Femoral Rotational Alignment in Total Knee Arthroplasty

**DOI:** 10.5704/MOJ.2103.012

**Published:** 2021-03

**Authors:** C Leelasestaporn, M Thuwapitchayanant, P Sirithanapipat, P Sa-ngasoongsong, P Ruengsilsuwit

**Affiliations:** 1 Department of Orthopaedic Surgery, Bhumibol Adulyadej Hospital, Bangkok, Thailand; 2 Department of Total Joint Replacement Center, Vejthani Hospital, Bangkok, Thailand; 3 Department of Orthopaedic, Ramathibodi Hospital, Bangkok, Thailand

**Keywords:** CAS in TKA, femoral component rotation, navigation in TKA

## Abstract

**Introduction:**

The aim of this study was to evaluate the reliability of the femoral component rotation on intra-operative data recorded in a computer-assisted navigation system (CAN-FRA) compared with the post-operative femoral component rotation observed on computed tomography (CT-FRA).

**Material and method:**

Computer-assisted total knee arthroplasty (TKA) or primary osteoarthritis of the knee was performed in 51 knees in 36 patients with a mean age of 69.51 years. All procedures were performed by a single surgeon using the same implant design. The intraclass correlation coefficient (ICC) was used to compare the intra-operative CAN-FRA with the post-operative CT-FRA. The angle between the anatomical epicondylar axis and the posterior condylar axis of the implant (CT-FRA) was measured at two separate timepoints by three observers who were blinded to the intra-operative CAN-FRA. Internal rotation was defined as rotation in the negative direction, while external rotation was defined as positive.

**Results:**

The mean intra-operative CAN-FRA was 0.1° ± 2.8° (range -5.0° to 5.5°). The mean post-operative CT-FRA was -1.3° ± 2.1° (range -4.6° to 4.4°). The mean difference between the CAN-FRA and the CT-FRA was -1.3° ± 2.2° (range -7.9° to 2.4°). The respective ICC values for the three observers were 0.92, 0.94, and 0.93, while the respective intra-observer coefficients were 0.91, 0.85, and 0.90. The ICC for the intra-operative CAN-FRA versus the post-operative CT-FRA was 0.71.

**Conclusion:**

This study shows that using a computer-assisted navigation system in TKA achieves reliable results and helps to achieve optimal positioning of the femoral component and rotation alignment correction.

## Introduction

Total knee arthroplasty (TKA) achieves good outcomes in the treatment of knee osteoarthritis. The success of TKA depends on multiple factors, including surgeon experience, surgical techniques, prosthesis design and implant material. In particular, the surgical technique is very important, as good soft tissue balance and appropriate positioning of the femoral and tibial components lead to a satisfactory outcome. Malpositioning of any component in TKA increases the risk of implant loosening, instability, patellar tracking (dislocation or subluxation), residual pain, and limited range of motion^1-8^. Appropriate implant positioning in TKA is very important, as this affects the functional outcome and survivorship^8,9^.

Although the appropriate implant positions and the identification of anatomical landmarks for implant insertion have been described, there are still issues with femoral rotation alignment, such as the attainment of the appropriate transepicondylar axis (the line connecting the medial and lateral epicondylar prominences)^10-12^. Studies comparing the component alignment attained using computer-assisted navigation (CAN) versus the conventional technique have found that CAN increases the accuracy of prosthesis alignment in TKA^4,13-24^, especially in patients with extra-articular deformity and tibial or femoral bowing^25,26^, and increases the accuracy of coronal alignment^20-24^. However, it has not been shown that femoral rotational alignment achieved via TKA performed with CAN is more accurate than that achieved in conventional TKA^11,15,17,27-29^. The aim of the present study was to evaluate the reliability of the femoral component rotation on intra-operative data recorded in a CAN system (CAN-FRA) compared with the post-operative femoral component rotation observed on computed tomography (CT-FRA).

## Material and Method

This was a single-centre prospective case series study in an academic hospital and was approved by our institutional ethical committee. The inclusion criteria were age > 50 years old, primary TKA, no history of previous fracture on the operated knee, and no sign of radiographic loosening of the knee implant. The exclusion criteria were deformity of the distal femur, and refusal to participate in the study. Following the study protocol, 36 consecutive patients (51 knees; 29 left and 22 right) with tricompartment osteoarthritis of the knee who underwent TKA performed with an imageless CAN system (CAN TKA) in our hospital from 1st January 2014 to 31st December 2015 were enrolled in this study. The demographic data are presented in Table 1. The mean patient age was 69.51 ± 7.30 years (range 51 to 81 years). Most patients were female (seven males, 29 females). The average patient height was 156.33 ± 5.67cm (range 146 to 170cm). The average weight was 63.14 ± 6.38 kg (range 43 to 92kg) and the average body mass index was 25.85 ± 8.20kg/m^2^ (range 19.11 to 36.85kg/m2). All procedures were performed by a single surgeon using the same implant design and CAN system [Scorpio NRG: The Stryker Navigation System Precision 4].

**Table I:** Patient characteristics

Parameter	Values
Age, year∇	69.51±7.30
Gender (male/female) 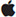	7/29
Height, cm∇	156.33±5.67
Weight, kg∇	63.14±6.38
Body mass index, kg/m2∇	25.85±4.20
Affected side (left/right) 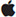	29/22

∇; data presented as mean±standard deviation

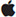
; data presented as number of cases

**Fig. 1:**
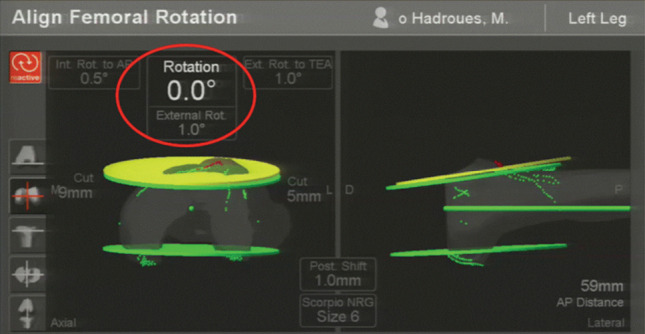
Screenshot of computer-assisted navigation showing the intra-operative registration record of the femoral component rotation (CAN-FRA).

With the knee in the flexed position, CAN was used to create a standard midline skin incision from about 5cm above the superior pole of the patella to the proximal part of the tibia. The registration process was done using an imageless CAN technique in which the centre of the femoral head was identified by moving and rotating the femur in a circular manner and the data was sent to the computer. The next step was the registration of the medial and lateral epicondyles for the determination of the transepicondylar axis, and the registration of the centre of the distal femur for the determination of the femoral axis and the anteroposterior axis (Whiteside’s line). The data of the femoral articular surfaces of the distal and posterior medial and lateral femoral condyles were then registered. The registered medial and lateral borders of the distal femur and the surface of the anterior cortex were used to estimate the required femoral component size. After the completion of the femoral registration process, the tibial registration was done. The centre of the tibial plateau was identified and the tibial axis was registered as the surface of the deepest part of the medial tibial plateau in varus deformity or the lateral tibial plateau in valgus deformity and the most prominent area of another tibial plateau, and the tips of the medial and lateral malleoli were identified to enable the CAN system to calculate the centre of the ankle. Pre-operative knee data (including the degree of deformity) were recorded, the soft tissue condition was evaluated to determine the correction of fixed deformity, and the knee kinematics were evaluated. After the registration process, the operation was started with the proximal tibial cut in accordance with the gap workflow technique. A freehand technique was used to cut the tibial bone in the best position perpendicular to the tibial axis in the coronal plane with a posterior tibial slope of 5°to 7° at a depth of 2 mm from the deepest part of the tibial plateau. After the proximal tibial bone was cut, the soft tissue was balanced in the flexion gap and extension gap by using a tension device to input the gap data into the computer. Femoral component sizing and positioning were then planned to achieve an acceptable mechanical axis and equal rectangular gaps. The computer-assisted navigated femoral rotation angle (CAN-FRA) (Fig. 1), final implant position, range of motion, mechanical axis, and joint stability were recorded. The no thumb test was used to identify the patella tracking problems during the component trial and after the final prosthesis insertion. All patients showed negative for the no thumb test in this study.

**Fig. 2:**
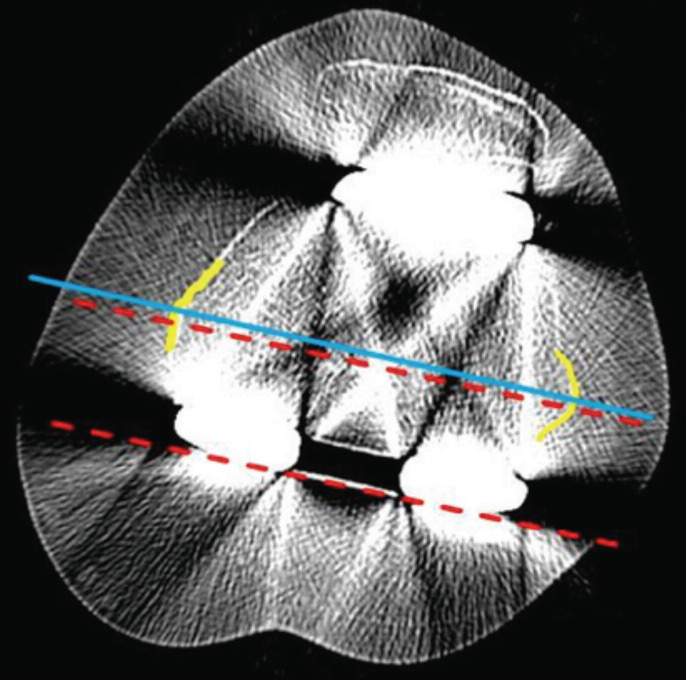
Computed tomographic image showing the measurement of the post-operative femoral component rotation (CT-FRA). The CT-FRA is the angle between the transepicondylar axis (blue line) and the posterior condylar axis of the femoral component (red dashed line).

The rotational alignment of the femoral component was measured on computed tomography using an axial view through the femoral component. The angle between the posterior condylar axes of the implants (CT-FRA) (red dashed line in Fig. 2) and the anatomical epicondylar axis, the line connecting the medial and lateral epicondylar prominences (blue line in Fig. 2), was measured at two separate timepoints (one month apart) by three observers who were blinded to the intra-operative CAN-FRA data. Internal rotation was defined as rotation in the negative direction, while external rotation was defined as rotation in the positive direction. Outlier was defined as the femoral component rotation angle between CAN-FRA and CT-FRA more than 3° either internally or externally^30^. SPSS version 20.0 software was used to calculate the intraclass correlation coefficient (ICC) values to estimate the inter-rater reliability of the analysis of the intra-operative CAN-FRA and the post-operative CT-FRA. In accordance with widely accepted research^31^, the inter-rater agreement was defined as poor for ICC values of less than 0.40, fair for ICC values between 0.40 and 0.59, good for ICC values between 0.60 and 0.74, and excellent for ICC values between 0.75 and 1.00.

## Results

**Fig. 3:**
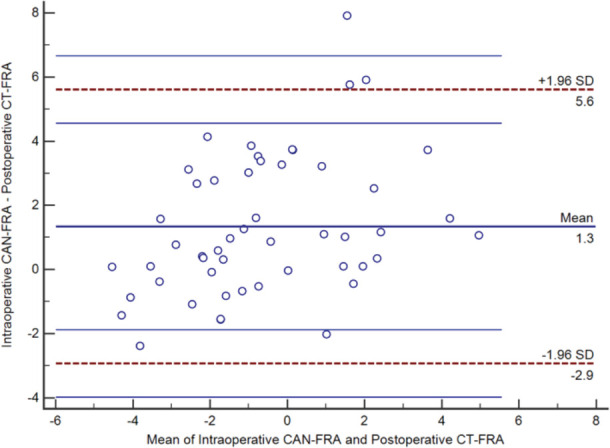
Graph showing the Bland-Altman plot of the mean difference between the intra-operative computer-assisted navigation data (CAN-FRA) and post-operative computed tomography measurements (CT-FRA).

**Fig. 4:**
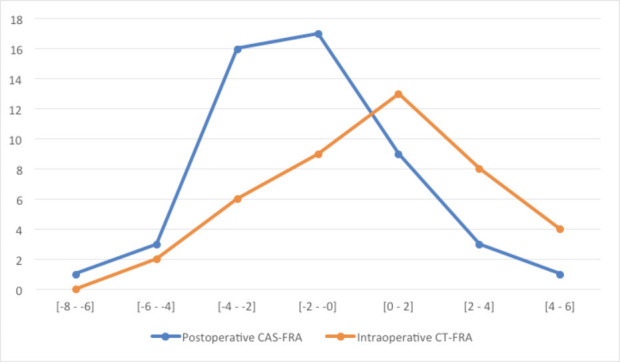
Line graph showing the difference in femoral rotation alignment between the intra-operative computer-assisted navigation data (CAN-FRA, red line) and the post-operative computed tomography measurements (CT-FRA, blue line).

The mean intra-operative CAN-FRA was 0.1° ± 2.8° (range -5.0° to 5.5°). The mean post-operative CT-FRA was -1.3° ± 2.1° (range -4.6º to 4.4º). Fourteen knees (27.5%) were identified as outlier due to the difference between CAN-FRA and CT-FRA more than 3°. Fig. 3 showed the mean difference between the CAN-FRA and CT-FRA was -1.3° ± 2.2° (range -7.9° to 2.4°). The respective ICC values of the three observers were 0.92, 0.94, and 0.93 (P < 0.01), while the respective intra-observer ICC values were 0.91, 0.85, and 0.90 (P < 0.01). The ICC for the intra-operative CAN-FRA versus the post-operative CT-FRA was 0.71 (P < 0.01). Fig. 4 shows the comparison femoral rotation alignment between the intra-operative CAN-FRA and the post-operative CT-FRA.

## Discussion

Several studies have shown that CAN improves the accuracy of implantation in TKA^4,13-24^ and increases the accuracy of coronal alignment of the lower limb^20-24^, but it remains controversial whether the femoral rotational alignment achieved via computer-assisted TKA is more accurate than that achieved via conventional TKA^11,15,17,27,28^. Moreover, a recent systematic review showed no evidence that CAN TKA decreases the number of femoral rotational alignment outliers^29^. Therefore, the aim of our study was to evaluate the reliability of rotational alignment of the femoral component in CAN TKA by comparing the intra-operative CAN-FRA with the post-operative CT-FRA.

In the present study, the CAN system [Precision 4 Stryker®] calculated the rotation of femoral component based on the transepicondylar axis and the anteroposterior axis (Whiteside’s line) to plan the implant positioning. Several studies have demonstrated that malrotation is affected by many factors, such as anatomical landmark registration errors, cementation, and errors in the cutting process^21,32-34^. In particular, one study showed that intra-observer errors occur because of errors in the registration of anatomical landmarks that affect the planning process^12^. A recent study found that incorrect registration during computer-assisted TKA leads to malpositioning of implants, and that the distal femoral epicondyles are the most difficult anatomic landmarks to register^35^. Therefore, the registration process is important in CAN TKA. As experienced surgeons make fewer errors during the registration process than inexperienced surgeons, the surgeries in our study were performed by a single high-volume surgeon with more than 10 years of experience with CAN TKA. A review of the literature retrieved only one study that attempted to determine the validity of intra-operative CAN alignment data compared with post-operative rotational alignment^36^. Similar to the present study, this previous study showed good intra and inter-observer reliability for post-operative CT measurements; however, in contrast to the present study, the previous study found that the intra-operative CAN data was significantly different to the post-operative rotational alignment^36^.

Our study also had some limitations despite of the favourable outcomes. First, the major factor of the success in this case series study is directly related to the experiences of the senior surgeon from the identification of bony landmarks and registration, precise bone cutting, and accurate implantation of the femoral component. Therefore, the present study did not demonstrate the accuracy of CAN by the beginners. However, the learning curve of beginners for CAN in TKA required only 16-20 cases to achieve the reproducibility as same as the experts^37,38^. Second, this study used only one surgeon due to the nature of a single-centre study. Therefore, multicentre prospective study with larger sample size is required to explore the accuracy of intra-operative CAN-FRA.

## Conclusion

The present findings showed that the rotational alignment of the femoral component achieved using a CAN system is reliable and helps to achieve the optimal positioning of the femoral component and rotation alignment correction in TKA.
